# Nature Studies

**Published:** 1910-01-08

**Authors:** 


### THE PRACTITIONER'S RELAXATIONS.

### III.—NATURE STUDIES.

By a MAJOR, R.A.M.C.

The First Meet—An Onslaught of Sea Gulls—Starlings and Snails—Mixed Flocks.

October blew out on a gusty Sunday night, but the first of November dawned fine for the opening meet, a great event here. I live near by the kennels. The wet and stormy night had not sent the foxes underground, or the stopping had been well done, for we found several foxes and had a good hunt. Whilst hounds were running in cover a woodcock sprang by them, circled over the heads of the field that waited outside, flew back into the cover again, was again disturbed, and again flew round over us. Then it went back over the cover and I saw it no more. This is the first cock I have seen this year.

The storms at the end of October brought seagulls in. A flock came down into a great penning behind my house. There was a herd of young horses in the penning. The unusual appearance and the strange clamour of the gulls frightened the horses and set them galloping. Hereabouts it is seldom gulls settle on the fields, though in wild weather I not infrequently see them flying over, and more rarely curlew. One day this autumn I saw a flock of curlew fly over at twilight. They came over my head, within gunshot, whistling loudly. Recent bright days have cheered the birds. On the seventh of November, about church-time, I heard a hedge-sparrow singing on the skirts of my domain. I found him sitting amongst the red berries of a hawthorn bush on the sunny and sheltered south side. In spite of a cold-blowing north wind he sang his song and repeated it.

On the downs now are vast flocks of starlings. On the sixth of November I was riding across the downs and went through an enormous flock or conglomeration of flocks. It is no exaggeration to say that there were on one stretch of rough down near a plantation known as Holme's Clump, many many thousands of these birds. They were very busy feeding; so busy that those in my line would hardly get away from my horse's feet. Every minute detachments hundreds strong rose and wheeled and settled to search for food again. I have shot starlings from flocks at this time of year and found the crops full of small snails. The number of snails amongst the down grass must be prodigious, for each bird eats dozens. It is amusing to watch the flocks. The birds walk along rapidly with a swaggering gait, peering and pecking this way and that, making little rushes as they spy a morsel of food. All moving in the same direction, they work the ground jealously, the hindmost birds rising, flying over those in the van, alighting just before them, and so taking the lead. This manœuvre is repeated again and again, so that the same birds are never for many seconds either first or last.

Feeding amongst the great flock I have mentioned was a covey of partridges. This was an accidental association that did not seem to the taste of either party; for I noticed that when a detachment of starlings wheeled and settled round and amongst the partridges the latter always got up and flew a little way and settled down on new ground. If they happened to settle where starlings were feeding they in their turn rose and wheeled and settled a little apart. This I saw many times repeated.

Excepting certain allied species mixed flocks of birds are seldom found. By the term mixed flocks I mean flocks made up of individuals of different species. Jackdaws and rooks flock together regularly and fly together to and from feeding-ground and roosting-place. So do wood-pigeons and stock-doves. Though flocks that on casual observation appear to be made up of several kinds of birds are daily to be seen in autumn and winter, especially when the weather is hard and the birds are driven to the water-meadows, these are not really mixed flocks according to my definition, but chance admixtures of separate flocks each of its kind that have chosen or have been driven by scarcity to the same ground. I think this even will be found to apply to the mixed flocks of finches now common in the high hedgerows, and to the flocks of redwings, fieldfares, and thrushes that feed together in the fields and meadows later in the year. Behind my house, on down that is enclosed to form a great penning, almost daily I see jackdaws, rooks, peewits, starlings, partridges, finches, and latterly fieldfares all feeding together; the fieldfares and finches chiefly on the thorn bushes. But on alarm the flocks go off each its own way without mixture of species. The rooks and jackdaws form a noisy crowd; the peewits, disciplined and orderly, rise and make off in a silent flock high in air; the starlings fly together in a pack, and usually come to earth again but little further on; the partridges spring with a whirr and cross the road to drop down in rough grass near a gravel pit. I have noticed with ducks on Indian jheels that though many different kinds may be resting on the same piece of water yet they are really in separate flocks, and on alarm fly round in flocks of a kind—teal, widgeon, pintail, mallard, gadwell, or as the kind may be, each sort keeping together as a rule.

